# Supplementary material for: Contribution of Oxidative Stress Induced by Sonodynamic Therapy to the Calcium Homeostasis Imbalance Enhances Macrophage Infiltration in Glioma Cells
Source: Cancers (Basel). 2022 Apr 18;14(8):2036. doi: 10.3390/cancers14082036 (PMC9027216; doi:10.3390/cancers14082036)
Supplement: Supplementary file 1 [file cancers-14-02036-s001.zip › cancers-1654333-supplementary/cancers-1654333-supplementary-figures.pdf]

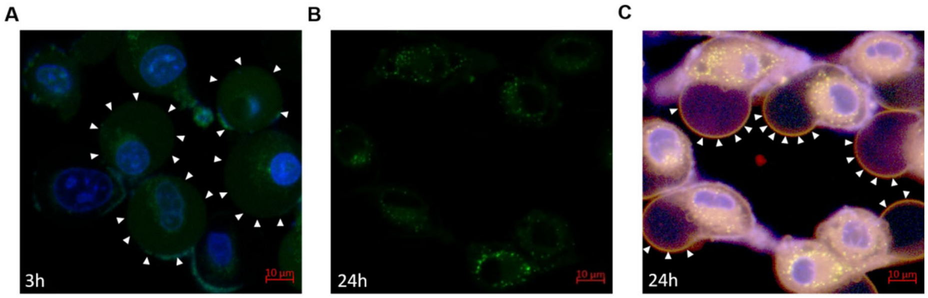


**Figure S1.** Morphological changes and Ca2+ staining after SDT treatment. (**A**) Free Ca2+ enters into the U251 cells, and the cell membranes become swollen (white arrow) due to the increased cell volume after 3 h. (**B**) Scattered distribution of intracellular Ca2+ after 24 h; (**C**) after Fig. S1 B was overexposed, the outline of the swollen cell membrane could be seen clearly (white arrow). Scale bar, 10 μm.

FABP4-U251

15%

3h-6h

β-actin

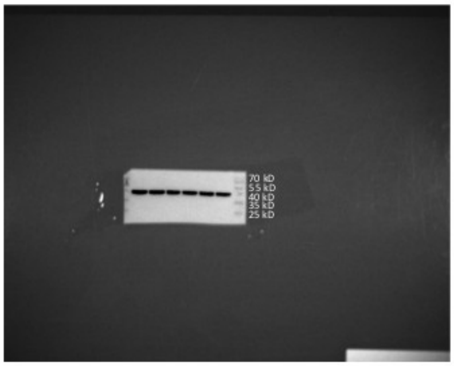




FABP4

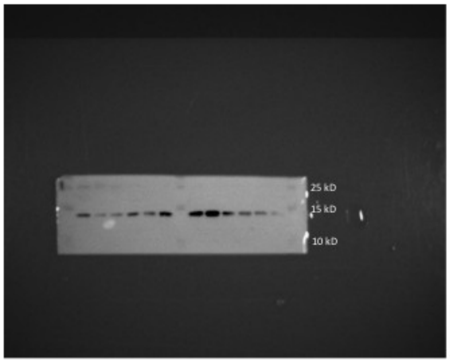




12h-24h

FABP4

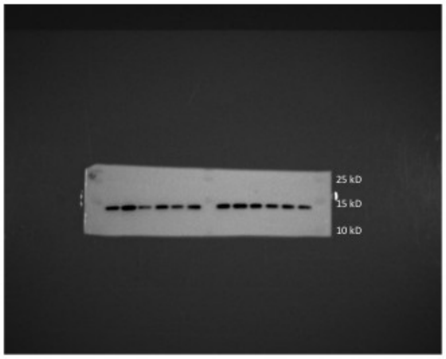

FABP4-U87

15%

3h-6h

β-actin

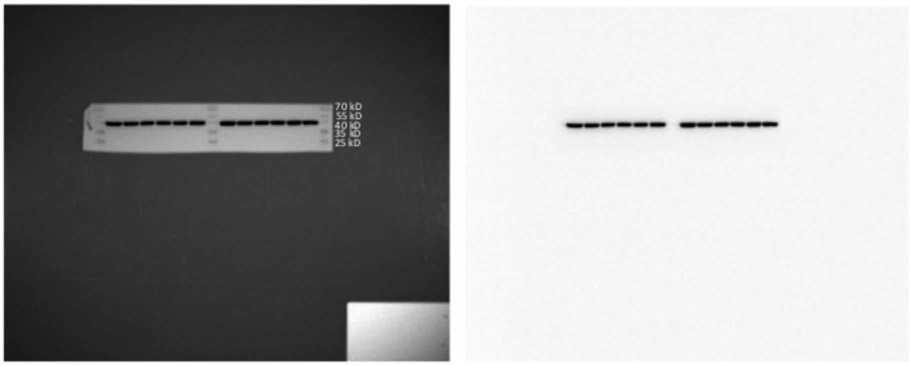


FABP4

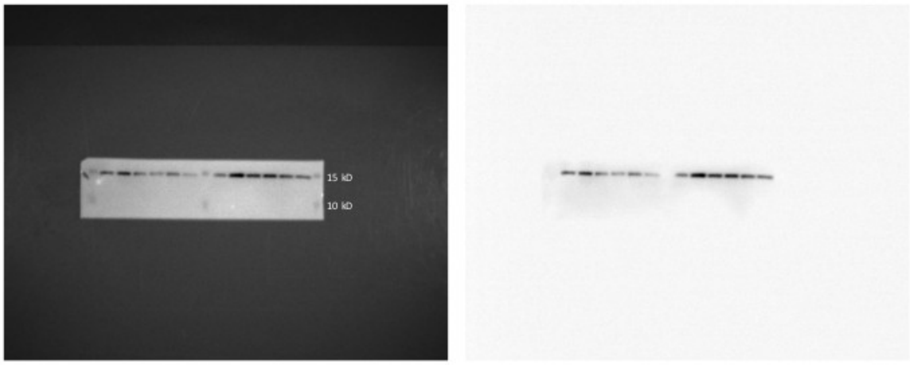


12h-24h

FABP4

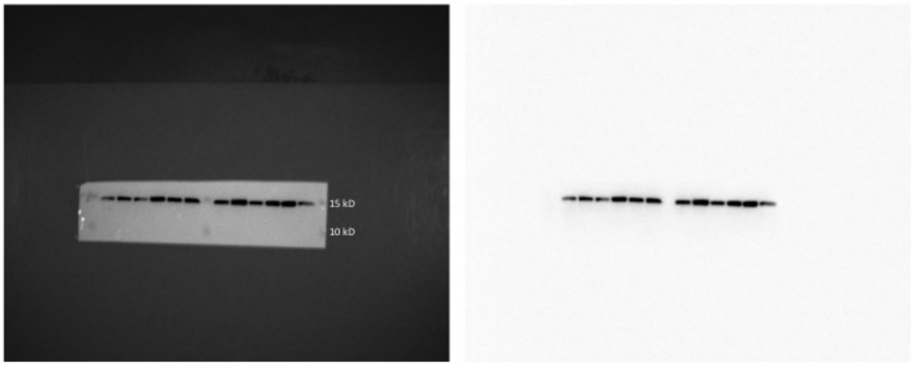


**Figure S2**: Uncropped WB figures.
